# ­­­Mechanical behavior of textile reinforced alkali-activated mortar based on fly ash, metakaolin and ladle furnace slag

**DOI:** 10.12688/openreseurope.14674.1

**Published:** 2022-06-17

**Authors:** Andres Arce, Lazar Azdejkovic, Luiz Miranda de Lima, Catherine G. Papanicolaou, Thanasis C. Triantafillou

**Affiliations:** 1 Department of Civil Enginnering, University of Patras, Patras, West Greece, 26222, Greece; 2 Department of Materials and Environment, Delft University of Technology, Delft, 2628, The Netherlands

**Keywords:** Alkali-activated materials, Bond, Geopolymer, Tensile strength, Textile reinforced mortar, SEM

## Abstract

The need for repair and maintenance has become dominant in the European construction sector. This, combined with the urge to decrease CO _2_ emissions, has resulted in the development of lower carbon footprint repair solutions such as textile reinforced mortars (TRM) based on alkali-activated materials (AAM). Life cycle studies indicate that AAM CO _2_ savings, when compared to Portland cement, range from 80% to 30%. Furthermore, in this study, recycled aggregates were considered with the aim to promote a circular economy mindset. AAM mortars formulation based on fly ash, ladle furnace slag and metakaolin were tested for compressive and flexural strength. Three out of all formulations were chosen for an analysis on the potential of these mortars to be used for TRM applications. Tensile and shear bond tests, combined with a concrete substrate, were executed as indicators of the TRM effectiveness. Scanning electron microscopy and chemical analysis based on energy dispersive X-ray spectroscopy were used to interpret the results and reveal the reasons behind the different level of performance of these composites. Results indicated that TRM based on high calcium fly ash are unsuitable for structural strengthening applications due to low bond between matrix and/or substrate and fibers. Metakaolin-based TRM showed good performance both in terms of tensile strength and bond capacity, which suggests potential as a repair mortar.

## Plain language summary

The paper explores the utilization of alkali activated technology to produce mortars, combined with carbon textile, for the development of textile-reinforced mortar. This composite is used for repair applications due to the enhanced shear, flexural, tensile, and confinement capacity that can be obtained through textile reinforced mortar application. Alkali-activated materials (AAMs) technology uses aluminosilicate rich products, in combination with an alkaline solution, to dissolve aluminum and silicate in the precursors and reorganize these particles to produce a cementitious binder. Through this technology, we aim to create alternative binders for repair applications that have potentially lower CO _2_ emissions than ordinary Portland cement (OPC) based mortars.

## Introduction

The construction sector provides 18 million jobs and contributes to 9% of the European Union’s GDP ^1^. Economic activities within this sector include new works, renovation, repair, and extension of buildings. From these economic activities, maintenance, repair and retrofitting have become more relevant in developed areas like Europe, while new construction is a major focus in developing countries, such as the ones located in Asia and Latin America ^2^. Lack of maintenance, and natural degradation mechanisms that affect existing buildings, have increased the importance of repair and structural upgrading. Therefore, it is imperative to develop solutions for the complex problems related to repair and retrofitting of existing buildings. One such technique is the application of reinforcing textiles in mortar (TRM) matrices, known as textile reinforced mortars, or fabric reinforced cementitious matrix composites. TRM composites combine high strength nonmetallic fibers (i.e., carbon, glass, basalt, polyphenylene bezobisoxazole), coated or uncoated, with inorganic matrices, such as cement, or the more recently developed alkali-activated binders. The mesh size of these textiles (or fabrics) typically varies from 8 to 30 mm, allowing the mortar to protrude through the openings and provide mechanical interlock between the matrix and the reinforcement ^3, 4^. The weight of these textiles is usually between 150 and 600 g/m ^2^ ^5^.

Textile reinforced mortars have become popular in the recent years due to several advantages over similar purpose techniques, like fiber reinforced polymers (FRP). Compared to FRP, TRM have the potential to provide a higher level of resistance when exposed to high temperatures, do not use high-cost epoxy resins, can be applied on wet surfaces, allow an effortless diagnosis of damage, and prevent potential conservation challenges related to trapped humidity due to their high porosity ^6, 7^. Due to the last two points, TRM have good potential as strengthening materials for historical masonry and concrete structures as well. TRM are also known for their high compatibility with masonry and concrete surfaces, subtracting the need of leveling with mortar existing in FRP applications, thus making them an easier applicable material compared to FRP ^8^. Textile-based composites had an increase in attention from the scientific community in the last two decades. Triantafillou and Papanicolaou ^9^, Akhoundi ^10^, Triantafillou and Bournas ^11– 13^ have done pioneering work on the strengthening of existing structures with TRM. Curbach and Jesse ^14^ studied the application of this technique on prefabricated elements. Triantafillou ^15^ made a comprehensive summary of the use of textiles in prefabricated elements and in retrofitting of existing concrete and masonry structures.

Similarly, AAMs have become popular in recent years due to the increased concern over climate change, and their potential to help the construction sector to decrease its CO _2_ emissions ^16^. Duxson *et al.* ^17^ reported a reduction of up to 80% in CO _2_ emissions by comparing OPC to various alkali-activated fly ash (FA) and metakaolin (MK) binders. Estimated CO _2 _savings calculated by life cycle analysis (LCA) vary from 80 % ^18^ to 30% ^19^, depending on the principles adopted for the LCA. Additional advantages of AAM over traditional cementitious materials are fire resistance ^20^ and high durability ^21^. The utilization of AAM in Australia ^22^, accounting for castings of a total of 70 000 tons of AAM concrete, is an evidence of their increased, but still low, acceptance in the construction sector. Despite their evident advantages, alkali-activated binders have great difficulties to enter the market due to research gaps, in addition to the lack of standards for these materials. It is, thus, necessary to create standards for alkali-activated mortars and concretes. The lack of regulations has huge implications for decision makers, specially engineers and owners, regarding risk and liability ^23^.

Recent studies have demonstrated the applicability of recycling industry byproducts in the construction sector, which can result in further reduction of CO _2_ emissions and preservation of natural resources ^24^. Furthermore, the ever-growing demand for construction supplies has led to a large-scale scarcity of natural resources and major sustainability concerns ^25^. It is within the objectives of this work to reincorporate waste or by-products into the value chain of the construction sector. It is known that solutions are needed to tackle the possible lack of raw materials in the future, to avoid the worst-case scenario in which natural resources are completely depleted. Therefore, in this research, electric arc furnace slag obtained from a steel processing company (Aeiforos S.A, Greece) is reutilized in the form of fine aggregates. Reutilization of byproducts helps reduce the size of landfills and some by-products even allow an enhancement of the properties of the materials in which they are incorporated ^26^. Previous studies on the replacement of limestone with electric arc furnace steel slag aggregates indicated an increase in compressive strength from 39 MPa to 53 MPa for industrial pavement concrete ^27^. In this study, the positive effect of these aggregates on AAM mortar formulation will be analyzed. 

This paper studies the potential utilization, for the first time, of textile reinforced mortars made of alkali-activated materials (TRAAM) based on mixtures of FA, ladle furnace slag (LFS) and MK. The proposed textile reinforced mortar combines the advantageous characteristics of alkali-activated mortars, recycling of by-products and the benefits provided by the textile reinforced mortar technique, to offer an innovative solution for repair and retrofitting procedures, in line with the current trends of recycling and reduction of carbon emissions. Materials and methods used to develop and test the specimens are shown in the next section, followed by the results of the mechanical characterization of mortar formulations along with the tensile and single-lap shear bond tests carried out on TRAAM specimens. A large experimental campaign was necessary to develop a mix design that would, not only fulfill mechanical strength requirements, but would also result in a mortar that is workable and can be used for practical applications.

## Methods

### Precursors

**Figure 1.**
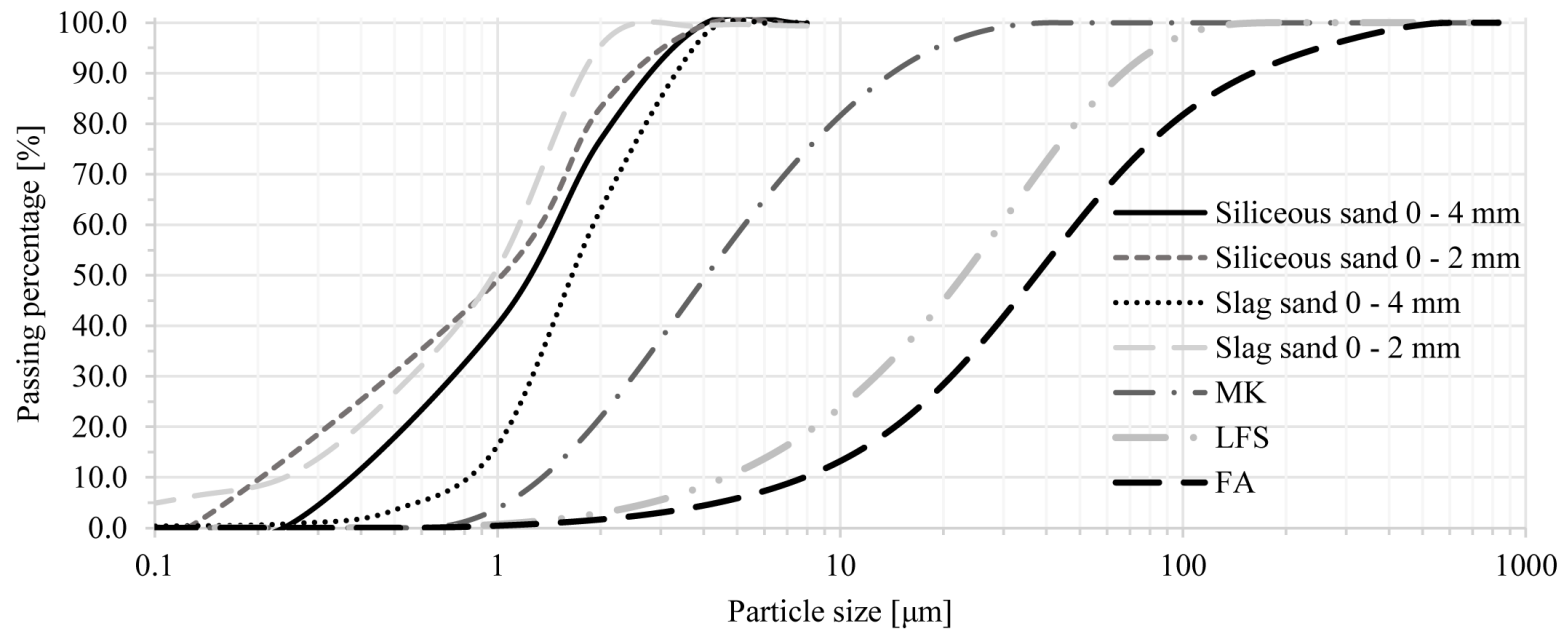
Particle size distribution of precursors and fine aggregate.

Three precursors were considered in the study: FA, LFS and MK. FA was obtained from a lignite electric power plant in Greece (DEI, Ptolemaida). The material was used as it was collected from the site. LFS was supplied by Aeiforos (the recycling segment of VIOHALCO company, Greece), and flash-calcined MK was manufactured by Imerys UK. Chemical composition was identified through X-ray fluorescence. Measurements were carried out at the Laboratory of Electron Microscopy and Microanalysis of the University of Patras. An amount of 1.8 g of dried ground sample was mixed with 0.2 g of wax (acting as binder) and was pressed on a base of boric acid to a circular powder pellet of 32 mm in diameter. Analyses were performed with a RIGAKU ZSX PRIMUS II spectrometer, which is equipped with Rh-anode running at 4 kW, for major and trace elements analysis. The spectrometer was equipped with the diffracting crystals LIF (200), LIF (220), PET, Ge, RX-25, RX-61, RX-40 and RX-75. Measured values are shown in Table 1. experimentally determined chemical compositions indicate a deficit in Al _2_O _3_ content for LFS and FA, which can be an indication of reduced reactivity in alkaline environments ^28, 29^. Laser diffraction particle size analysis of precursor materials was also carried out, with outputs presented in Figure 1. MK is the finest material of the precursors with a D _50_ of 4.68 µm, while LFS and FA displayed broader particle size distributions with D _50_ of 26.54 µm and 42.82 µm, respectively.

**Table 1.** Chemical analysis of precursors, by weight *.

Precursor	SiO _2_ (%)	Al _2_O _3_ (%)	CaO (%)	Fe _2_O _3_ (%)	Na _2_O (%)	MgO (%)	P _2_O _5_ (%)	K _2_O (%)	TiO _2_ (%)	MnO (%)	LOI-Flux (%)
Metakaolin	47.84	45.61	0.30	0.39	0.32	0.22	0.08	0.19	1.42	-	0.17
Fly Ash	29.25	13.08	27.09	3.17	0.35	3.56	0.40	1.09	0.64	0.01	3.10
Ladle Furnace Slag	20.55	3.52	41.38	1.40	0.10	3.70	0.01	0.05	0.24	0.49	6.82

*Only detectable chemical compounds listed.

### Chemical activators and compounds

Sodium and/or potassium-based activators were used for all mortars produced. Sodium hydroxide (98% reagent grade) was used in different proportions in combination with sodium silicate (SiO _2_/Na _2_O modulus of 3.4, 80% purity), aiming at studying the influence of Si/Na on properties of fresh and hardened mortar. Potassium carbonate was used in one mix as a replacement for sodium-based activators due to its lower carbon footprint. Potassium hydroxide (90% purity) was combined with potassium silicate in different proportions and used for MK-based TRAAM composites. In both cases, pellets were dissolved in water and further mixed with liquid silicate solutions. Solutions were left to cool down to room temperature before mixing with precursors. A polycarboxylate-based superplasticizer was also used in the mixes containing FA.

### Fine aggregates

Three different fine aggregates were used in mortar mixes: siliceous sand and electric arc furnace slag sand, in two fractions, namely from 0 to 4 mm and 0 to 2 mm; and marble stone filler, which was used in order to achieve better packing density in trial mixes. This marble stone powder is an inert calcium carbonate-based material with a mean particle size of approximately 4 μm and a maximum particle size of 10 μm. The particle size distribution curves of all sands are shown in Figure 1.

### Textile reinforcement

A balanced (equal quantity of fibers in two orthogonal directions) uncoated carbon fiber textile was selected for the study (Mapegrid C170, manufactured by MAPEI S.p.A). Mass of the textile was 180 g/m ^2^. The distance between center lines of rovings was chosen to be 10 mm. The density of the fiber was 1839 kg/m ^3^. Nominal thickness, as reported by the manufacturer, was taken as 0.048 mm. The average peak stress, associated strain and modulus of elasticity of this product were previously measured by Kapsalis ^30^ and reported to be 2200 MPa, 0.0136 and 195 GPa respectively . The coefficient of variation was of 7.0%, 10.2% and 10.1% in the same order. Strips of textile were subjected to tensile load until failure to characterize the stress-strain behavior of the product. Sample width was approximately 70 mm incorporating 7 rovings. Metallic plates (tabs) were bilaterally bonded at both ends of the textile strip to distribute gripping stresses to the whole mesh and prevent early failure of the textile. The free length of the specimen was equal to 250 mm. The test was executed at a displacement-controlled rate of 0.02 mm/s. An optical extensometer was used to measure the strain in the textile. The maximum tensile strength provided by the manufacturer represents the ultimate capacity of the fiber at the highest level of efficiency. This capacity is often not achieved in real applications, as it is almost impossible to activate all rovings and filaments of fibers simultaneously. The failure of textiles occurs gradually as the fibers are not loaded equally, due to their relative slippage within a roving or unequal initial tension in different rovings.

## Alkali-activated mortars for use as matrices in TRM

### Mix designs

For FA/LFS mixes, a “one factor at a time” (OFAT) experimental campaign was designed to study the influence of the molar ratio of silicate (SI) to sodium (NA), the FA-to-LFS weight ratio, the slag-to-siliceous sand weight ratio, and the total amount of chemical activators, binders, and aggregates. The test campaign focused on studying the sensitivity of the mechanical properties for each mix (compressive and flexural strength), as a result of the variation of its components. The starting point was a mix - here called “reference” - based on the combination of FA, LFS, slag and silicious sand, which was extensively studied by Papayianni *et al.* ^31– 33^. Mix formulations for 1 m³ of mortar for each mix design based on FA and LFS are shown in Table 2. All mixes were prepared using fine aggregate 0 – 4 mm, in size. Mix nomenclature is based on a two-code system: XX_YY, where XX corresponds to the variable changed and YY its relative value (HI: high, ME: medium, LO: low & ZERO). XX abbreviations are as follows: SI: silicate-to-sodium ratio, FA: FA-to-LFS ratio, SS: silica sand-to-slag sand ratio, ACT: total amount of chemical activators, BIN: total amount of binders and AGR: total amount of aggregates. The assessment of all FA/LFS mixes presented in Table 2 ^34^, in terms of mechanical properties, (see the next section) showed that the SI_HI mix achieved an improvement in both compressive and tensile strength without compromising workability compared to the “reference” mix and all other formulations. Subsequently, it was decided to design and produce two variations of this mix: 1) FA_NA, which was only different in the gradation of sand used (0 – 4 mm was replaced for 0 – 2 mm, for improved mortar penetrability through the mesh openings in TRAAM composites), and 2) FA_KC, identical to FA_NA in terms of constituent materials, except for the replacement of the sodium-based activators for a potassium carbonate one, as described in Table 3.

**Table 2.** Mix formulations based on fly ash and ladle furnace slag precursors (quantities expressed per 1 m ^3^ of mortar).

Mix code	Solution * /binder ratio	Super plasticizer (kg)	Sodium hydroxide (kg)	Sodium silicate (kg)	Water ** (kg)	Fly ash (kg)	LFS (kg)	Binder *** (kg)	Siliceous sand (kg)	Slag sand (kg)
Reference	0.53	20.6	24.8	12.6	328.2	548.4	137.1	722.9	719.8	308.5
SI_HI	0.53	20.6	22.9	14.5	328.2	548.4	137.1	722.9	719.8	308.5
SI_ME	0.53	20.6	28.5	8.9	328.2	548.4	137.1	722.9	719.8	308.5
SI_LO	0.53	20.6	31.9	5.5	328.2	548.4	137.1	722.9	719.8	308.5
FA_LO	0.53	20.6	24.8	12.6	328.2	137.1	548.4	722.9	719.8	308.5
FA_ME	0.53	20.6	24.8	12.6	328.2	342.8	342.8	722.9	719.8	308.5
FA_HI	0.58	56.1	24.3	12.4	322.6	606.4	67.4	710.5	707.5	303.2
SS_ZERO	0.53	20.6	24.8	12.6	328.2	548.4	137.1	722.9	0.0	1028.3
SS_LO	0.53	20.6	24.8	12.6	328.2	548.4	137.1	722.9	308.5	719.8
SS_ME	0.55	47.4	40.6	20.7	299.6	541.4	135.3	738.0	710.5	304.5
ACT_ME	0.50	20.6	57.8	29.4	278.4	548.4	137.1	772.7	719.8	308.5
ACT_HI	0.57	86.3	72.0	36.6	245.4	531.1	132.8	772.5	697.0	298.7
BIN_LO	0.59	21.3	25.6	13.0	339.3	510.2	127.6	676.4	744.1	318.9
BIN_ME	0.53	70.1	23.0	11.7	305.3	586.6	146.7	768.1	669.5	286.9
BIN_HI	0.50	86.6	21.8	11.1	289.2	628.2	157.0	818.2	634.2	271.8
AGR_LO	0.56	41.5	26.5	13.5	350.9	586.4	146.6	772.9	654.2	280.4
AGR_ME	0.55	31.7	22.9	11.7	303.9	507.8	127.0	669.4	766.5	328.5
AGR_HI	0.55	29.7	21.5	10.9	284.6	475.5	118.9	626.7	811.3	347.7

**Solution stands for alkaline solution which comprises water, sodium hydroxide and sodium silicate **Water stands for the sum of the water from silicate solutions and additional water used ***Binder stands for the sum of FA, and the dry part of the chemical activators

**Table 3.** Mixture formulations based on previous mix SI_HI (quantities expressed per 1 m ^3^ of mortar).

Mix code	Solution */ binder ratio	Super plasticizer (kg)	Sodium hydroxide (kg)	Sodium silicate (kg)	Potassium carbonate (kg)	Water* * (kg)	Fly ash (kg)	LFS (kg)	Binder *** (kg)	Siliceous sand (kg)	Slag sand (kg)
FA_NA	0.53	20.6	22.9	14.5	0.0	328.2	548.4	137.1	722.9	719.8	308.5
FA_KC	0.53	20.6	0.0	0.0	37.4	328.2	548.4	137.1	722.9	719.8	308.5

**Solution stands for alkaline solution which comprises water, sodium hydroxide and sodium silicate **Water stands for the sum of the water from silicate solutions and additional water used ***Binder stands for the sum of fly ash, ladle furnace slag and the dry part of the chemical activators

A second round of tests studied the development of AAM mortars based on MK. Mix formulations are listed in Table 4. Since the single-precursor trial mix made of MK (mix MK_ref, in Table 4) was not able to reach the desired level of workability due to high viscosity (the flow table test resulted in 130 mm of spread), the focus of the research for this precursor moved to blended mixes. Both binary (MK/LFS) and ternary (MK/FA/LFS) precursor blends were studied. Nomenclature is based on MK_iBXj system, in which MK is common for all mixes; “iB” differentiates the number of precursor components in blends (i=1,2); “X” refers to the alkali metal used as activator; while “j” denotes the MK-to-LFS ratio (1-high, 2-medium, 3-low). Due to the highly viscous nature of MK/LFS-blend mixes, workability characteristics were mainly assessed by visual observation, flowability rating (flow table tests according to EN 1015-3 ^35^ on selected mixes) and applicability to horizontal and vertical surfaces. Chemical activators used for the activation of the precursor(s) were: sodium silicate solution (M _s_ = 3.4, dry content approximately 34.4%) with sodium hydroxide (mixes MK_2BNj, 2B standing for the binary precursor blend); potassium silicate (M _s_ = 1.6, dry content approximately 45%) with potassium hydroxide (mixes MK_2BKj and mix MK_3BK, 3B standing for the ternary precursor blend); and potassium silicate with sodium hydroxide (mix MK_3BNK). Siliceous river sand 0 – 2 mm was used in all mix trials. Additionally, in some mixes, marble powder filler was used for the enhancement of the packing density. None of the mixes contained superplasticizer. More information on mix design strategy for MK-based mixes is provided in the next section.

**Table 4.** Mixes based on metakaolin, fly ash and ladle furnace slag blends (quantities expressed per 1 m ^3^ of mortar).

Mix code	Solution */ binder ratio	Potassium hydroxide (kg)	Sodium hydroxide (kg)	Sodium silicate (kg)	Potassium silicate (kg)	Water ** (kg)	Metakaolin (kg)	Fly ash (kg)	LFS (kg)	Binder *** (kg)	Siliceous sand (kg)	Marble filler (kg)
MK_ref	0.93	0.0	53.4	462.7	0.0	303.5	338.1	0.0	0.0	553.5	1245.8	0.0
MK_2BN1	0.75	0.0	109.6	433.8	0.0	427.3	473.7	0.0	182.6	915.2	1118.7	0.0
MK_2BN2	0.69	0.0	95.6	314.8	0.0	382.4	425.9	0.0	222.2	849.0	1101.9	0.0
MK_2BN3	0.79	0.0	103.6	328.2	0.0	474.3	380.0	0.0	276.3	872.8	1115.7	0.0
MK_2BK1	0.79	118.2	0.0	0.0	413.2	455.6	473.7	0.0	182.6	960.5	1118.7	0.0
MK_2BK2	0.68	104.8	0.0	0.0	303.7	370.2	431.5	0.0	224.9	897.8	1118.7	0.0
MK_2BK3a	0.77	108.6	0.0	0.0	379.3	436.8	436.8	0.0	218.4	934.5	1034.5	114.9
MK_2BK3b	0.75	105.5	0.0	0.0	385.1	417.1	436.8	0.0	218.4	934.0	1034.5	80.5
MK_3BK	0.77	103.5	0.0	0.0	362.0	459.1	415.0	100	160.0	941.4	980.0	60.0
MK_3BNK	0.80	0.0	81.9	0.0	370.5	469.9	424.8	61.4	163.8	898.6	1003.1	0.0

**Solution stands for alkaline solution which comprises water, sodium hydroxide and sodium silicate **Water stands for the sum of the water from silicate solutions and additional water used ***Binder stands for the sum of fly ash, ladle furnace slag and the dry part of the chemical activators

For all mortars, dry materials were mixed by hand until homogenized. The activators were dissolved in water; the superplasticizer, when added, was combined with the chemical solution to form part of the liquid phase (solid activators dissolved in water and superplasticizer), which was added to the dry materials followed by a hand mixing procedure before using the electrical mixer. The materials (for a 1.5 kg batch of mortar) were mixed with an automatic standard mortar mixer Matest S.A. model equivalent to E093N (in compliance with EN196-1) ^36^ at a low speed for 2 minutes and at a fast speed for 5 minutes. A similar procedure was followed for larger batches of mortar produced to cast tensile test specimens (described ahead), for which the table mixer was replaced by an electric power mortar mixer supplied; in these cases, the mixing duration was extended to 7–10 minutes. Specimens were kept under moist burlap covers until testing. Heat curing was not employed for any specimen.

### Fresh- and hardened-state properties

The consistency of fresh mortar samples was assessed by means of a flow table test, and was expressed in terms of the mean diameter of the mortar sample after jolting the table for 15 times (as per EN 1015-3 ^28^). Mortar prisms measuring 40 × 40 × 160 mm were cast and tested after seven days for flexural and compressive strength, according to EN 1015-11 ^37^. This age was deemed adequate for the mechanical characterization of the mortars, and their comparative evaluation aimed at the selection of optimum ones.

**Figure 2.**
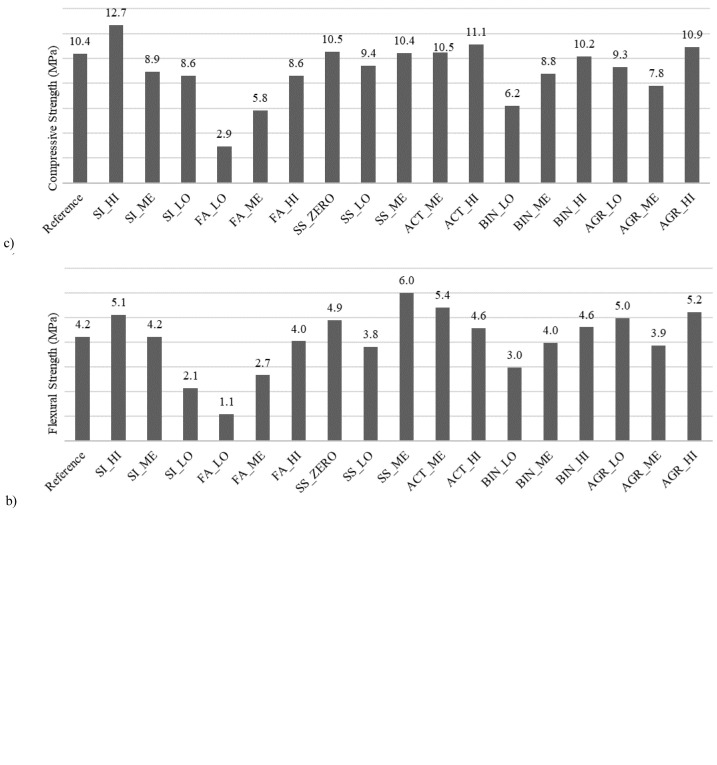
Fly Ash (FA) based mortars: **a**) Flow spread, **b**) flexural strength and **c**) compressive strength; Metakaolin (MK) based mortars: **d**) Flow spread, **e**) flexural strength, **f**) compressive strength.

The development of a mortar to be used as matrix in textile reinforced composites, applied as a seismic retrofitting material, requires three main characteristics: minimum flexural strength in the order of 5 MPa, spread value (evaluated by the flow table test as per EN 1015-3 ^28^), and adequate bond capacity with existing substrates (so that shear bond failure of TRAAM/substrate joints is not governed by the TRAAM/substrate interface shear strength). For formulations based on FA, the “reference” mix provided 4.21 MPa flexural strength after seven days, a spread of 185 mm and a compressive strength of 10.4 MPa. 17 mix variations were prepared and presented in Table 2 aiming to improve these values. Figure 2 (.a, .b, .c) shows the values of flexural strength, flow spread and compressive strength for all mix designs reported in Table 2. 

Mix SS_ZERO showed higher flexural strength, spread and compressive strength than the “reference” mix, which indicates that even complete substitution of natural siliceous sand with fine aggregates deriving from industrial by-products (slag sand) is possible, and could even be recommended. Mixes SI_HI, SS_ME, ACT_ME, ACT_HI, AGR_LO and AGR_HI exhibited an improvement in flexural strength compared to the “reference” mix. While all previously mentioned mixes showed an increase in flexural strength, they also resulted in a decrease in spread and, hence, in lower workability which was undesirable. One exception was observed for formulation SI_HI, as it had a similar workability to the reference mix. Based on these results, mix SI_HI was selected as the optimum mix from this series of batches, as it showed an improvement in flexural and compressive strength at a zero loss of workability. Mix SI_HI was formulated with fine aggregates with a size of 0–4 mm. As previously mentioned, its FA_NA variation (identical to SI_HI in terms of all constituent materials proportions) comprised fine aggregates with a size of 0–2 mm, with the aim to facilitate mortar protrusion through the grid openings and, thus, enhance the textile-to-mortar bond conditions. Another variation (mix FA_KC), was identical to mix FA_NA, with the only difference being the replacement of sodium-based activators with a potassium carbonate one. Both mixes (FA_NA and FA_KC, Table 3) were assessed strength-wise at an age of seven days. The flexural strength of mix FA_NA reached 4.1 MPa with no change in spread, but a considerable drop in compressive strength (from 12.7 to 7.4 MPa). The flexural strength of mix FA_KC decreased to 3.8 MPa, spread decreased to 160 mm and the compressive strength dropped (from 12.7 to 10.6 MPa). Although both mixes resulted in inferior performance indices in regard to the reference mix, it was decided to use them in order to proceed to TRAAM production as: (i) their aggregate skeleton was of a higher fineness than that of the “reference” mix, and (ii) their long-term strength characteristics would be sufficient to ensure adequate mortar/textile composite action.

The mechanical properties of the MK-based series of mortar batches (MK_ series) were assessed seven days post-production and are reported in Figure. 2 (.d, .e, .f). The mortar mix based on MK as single precursor, mix MK_ref, showed satisfactory flexural and compressive strength, but substandard workability. Therefore, LFS was introduced to a new set of mix design trials. However, mortars with blended precursors that showed satisfactory mechanical properties had a fluid consistency with a spread value exceeding 230 mm. Spread was retained for 30 minutes after mixing before the onset of workability decline. Sand-to-precursor mass ratio was kept close to 1.7 which provided an inert skeleton that was adequate enough to control shrinkage rate, but at the same time kept the water demand at an acceptable level (H _2_O/(K _2_O+N _2_O) molar ratio was kept below 16). The MK-to-LFS ratio was investigated through mixes MK_2BNj and MK_2BKj. Due to the presence of excessive drying shrinkage in mixes containing high amounts of LFS, the maximum quantity of the latter was set at 33% of the total precursors’ weight. Marble stone filler was introduced as a replacement for 5 to 10% of siliceous sand. Potassium-based mixes exhibited better workability characteristics compared to sodium-based ones (which are thixotropic in nature), due to the low viscosity of potassium silicate (20 mPa·s at 20°C). Ternary mixes were prepared and tested by adding FA to the MK/LFS blend, activated by potassium-based (MK_3BK) and a combination of potassium and sodium-based alkaline solution (MK_3BNK). Even though the round-shaped FA particles significantly improved the workability of mortars, large particle size and high CaO content of the FA increased the water demand significantly, resulting with the decrease of flexural and compressive strength of mortar mixes. Furthermore, the addition of FA significantly affected the workability retention properties thus, initiating flash-setting in cases where larger amounts of FA were added. Due to this reason, the further investigation of ternary mixes was abandoned while the setting time analysis is beyond the scope of this paper. Finally, considering the results of the flexural and flow table tests of all mixes, the mortar denoted as MK_2BK3b was selected as the most suitable to be used as a matrix material in TRAAM strengthening systems. Even though the initial consistency of MK_2BK3b mix was similar to all other binary mixes activated with potassium-based activators, after 10 minutes of pot life, this mix was easier to apply on a vertical surface compared to MK_2BK2 and MK_2BK3a. Mix MK_2BK3b was recorded to have a continuous increase in both flexural and compressive strength throughout time (7 MPa and 54 MPa, respectively measured at 180 days of age), and a significantly longer pot life compared to MK_3BKN.

## Textile-Reinforced Alkali-Activated Mortars

In order to investigate the mechanical behavior of TRAAM composites, tensile and single-lap direct shear bond tests were carried out. The load was automatically recorded by the load cell connected to the servo-hydraulic testing machine. Displacements were obtained through a video-extensometer RTSS-HR purchased from Limess GmbH. The gauge length of the tensile specimens was taken equal to the total free (not clamped) length of the specimen. A similar approach was followed for the bond test, where the whole bonded substrate was included in the strain measurements.

### Tensile tests

Mixes FA_NA, FA_KC ( Table 3) and MK_2BK3b ( Table 4) were selected to produce TRAAM tensile coupons by combining the mortars with two layers of a carbon fiber mesh. Specimens were cured for 28 days before testing, wrapped in wet cloths and kept under plastic covers to prevent humidity loss. The tensile test followed the provisions set in AC434 standard ^38^. Dimensions were chosen to both fulfill the requirements of AC434 and be representative of typical on-site strengthening applications with TRM jackets; commonly comprised of at least two layers of textile. The TRAAM specimens had a thickness of 11 mm, consisting of two layers of a carbon fiber textile and three layers of AAM mortar. The two external mortar layers were of a thickness equal to 4 mm, whereas the middle one (keeping the two textile layers apart) was 3 mm-thick. The fiber mesh was sized so as to extend from both sides of the specimen for at least 100 mm; the purpose was to allow for manual tensioning of the textile ensuring the fibers’ alignment and activation at early stages of tensile loading.

The specimens were furnished at both ends with 3 mm-thick, 100 mm-long, full-width steel tabs (two at each side, one glued at each face). The tabs featured a hole to facilitate fitting to the shackle of the clevis-type end fixtures which – in turn – were firmly connected to the universal testing machine heads. The latter was a servo-hydraulic testing machine with a capacity of 250 kN. Tests were run in a displacement-controlled mode at a rate of 0.002 mm/s. Displacements were recorded by a video-extensometer set to monitor the relative displacement between two horizontal lines on the specimens symmetrically drawn in respect to their mid-span. The video-extensometer was synchronized with the load-cell’s outputs.

Stresses were computed by dividing the actuator load F by the cross-section area of the fibers running along the loading direction, as per Equation 1:

$$\sigma =\frac{F}{A_{f}}\;(1)$$

$$A_{f}=2\times t_{nom}\times b\;(2)$$

where: σ is the stress in the textile; *F* is the load applied by the actuator; *A _f_* is the area of load-aligned carbon fibers per layer of textile; *t _nom_* is the design nominal thickness of the textile; *b* is the width of the specimen.

The experimentally obtained maximum textile stress (2200 MPa) was compared to the maximum stress reached in the composites (textile reinforced mortars) to calculate an exploitation ratio (bare textile strength over composite strength) which is reported in Table 5 along with the peak (maximum) stress and respective strain of each specimen and the average values for each test group (i.e. for each type of matrix).

**Table 5.** Tensile test results of coupons made of textile reinforced alkali-activated mortars.

Specimen ID	Peak stress (MPa)	Strain at peak stress (%)	Exploitation ratio	Average stress (MPa)	Coefficient of variation (%)	Average strain at peak stress (%)	Coefficient of variation (%)	Mean initial modulus of elasticity (MPa)
T_FA_NA_1	399.9	0.23	0.18	464.4	17.0%	0.29	27.2%	2702.3
T_FA_NA_2	441.0	0.26	0.20					
T_FA_NA_3	552.3	0.38	0.25					
T_FA_KC_1	592.2	0.39	0.27	649.4	12.9%	0.43	10.5%	3139.9
T_FA_KC_2	610.3	0.43	0.28					
T_FA_KC_3	745.8	0.48	0.34					
T_MK_2BK3b_1	1265.9	0.87	0.58	1271.3	1.0%	0.86	3.3%	13580.7
T_MK_2BK3b_2	1261.7	0.89	0.57					
T_MK_2BK3b_3	1286.3	0.83	0.58					

**Figure 3.**
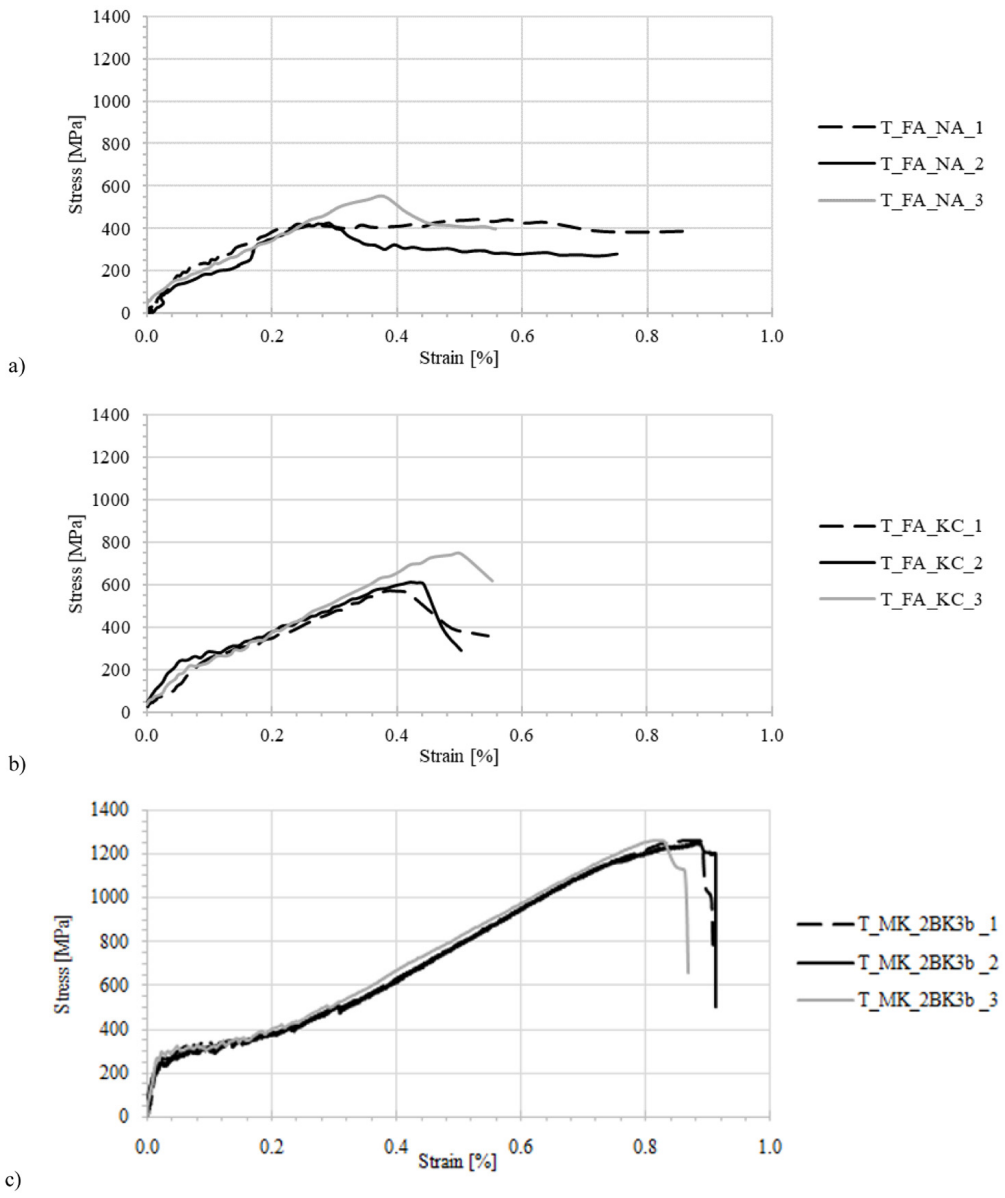
Stress strain curves for alkali-activated material specimens under uniaxial tensile loading. Mortar formulations: **a**) FA_NA, **b**) FA_KC, **c**) MK_2BK3b.

**Figure 4.**
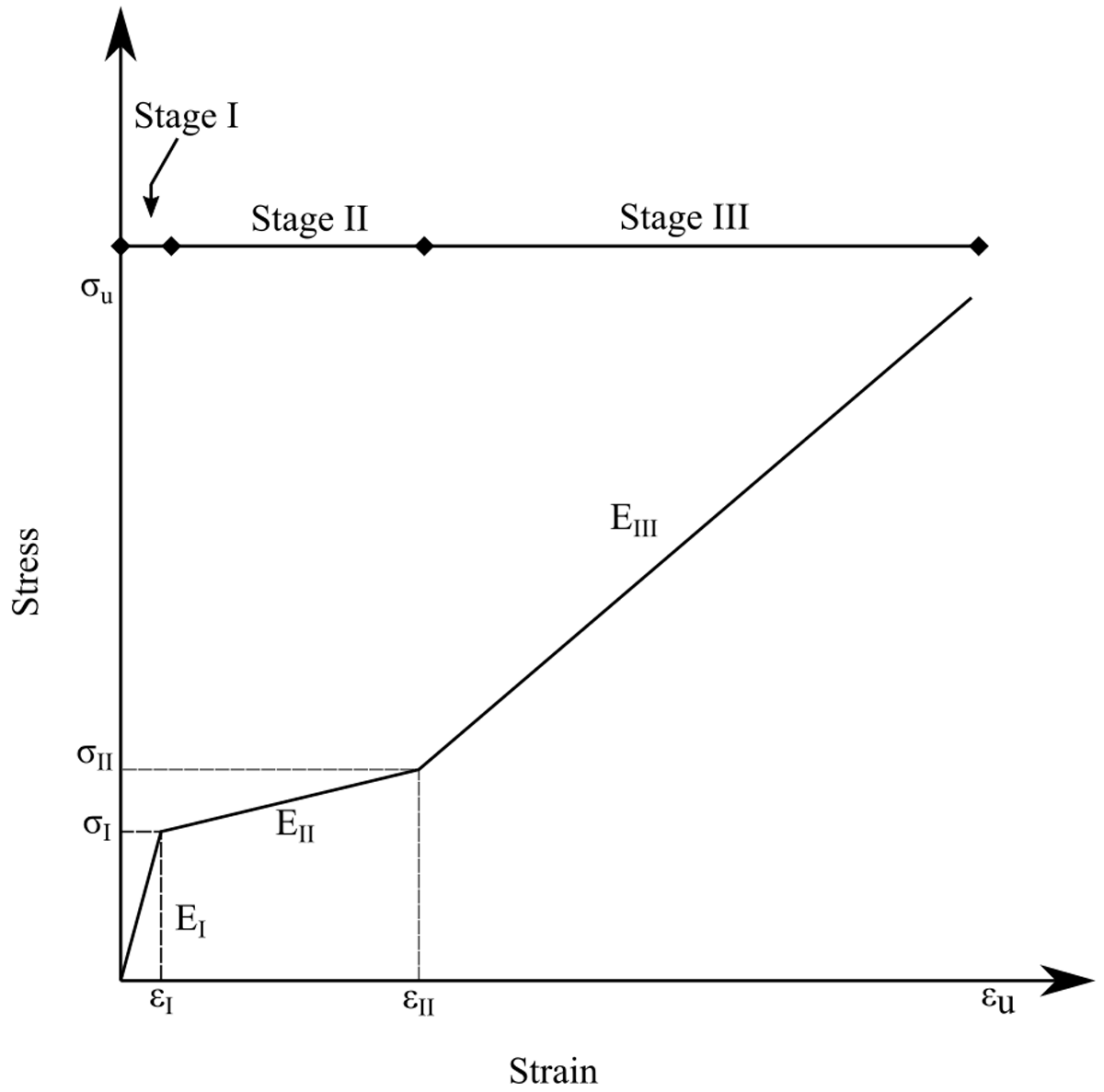
Typical stress-strain response of a textile reinforced mortar specimen.

The stress-strain curves from all TRAAM tensile test specimens are given in Figure 3. Typically, the response of a TRM specimen undergoing tensile loading develops in three ^39– 41^ or two ^42^ quasi-linear stages. TRM produced with alkali activation of FA/LFS blends and tested in this study differ considerably from this well-accepted stress-strain relationship. TRAAM based on formulations FA_NA and FA_KC developed a clearly elastic initial stress-strain behavior until a crack was formed at a very low strain (between 0.02–0.04%). This behavior corresponds to Stage I in Figure 4. The expected second stage, corresponding to a partial composite action between matrix and fibers that would allow the widespread formation of cracks in the matrix, was barely present in these two types of TRAAM. Instead, the first (and only) crack widened as more load was applied. Most of the strain of the specimen was recorded post-peak (in Stage III) due to the opening of this single crack.

The average tensile strength of the sodium hydroxide/sodium silicate-activated mortar specimens (T_FA_NA series) was equal to 464 MPa, accounting for a mean efficiency ratio of 0.22, the lowest of the three composites. It is likely that a low mortar-textile bond prevented the textile from being activated and instead it slipped from the mortar ( Figure 3.a). More details about the textile-mortar bond for all types of TRAAM are provided in the following section. The average tensile strength of specimens prepared with potassium carbonate as activator (T_FA_KC_ series Figure 3.b) was 649.4 MPa, resulting into a mean exploitation ratio of 0.30.

MK_2BK3b -based specimens performed considerably better. Stress-strain diagrams consist of three parts: the uncracked stage, the multiple cracking stage and the strain-hardening one ( Figure 3.c), which resemble TRM material models based on OPC matrices ^43^. The behavior of T_MK_2BK3b specimens subjected to axial tension was satisfactory due to the presence of a strong mechanical interlock between the matrix and the textile. The latter was manifested by the formation of a dense network of distributed cracks along each tested specimen and the presence of a strain hardening effect ( Figure 3.c). The exploitation ratio for this type of textile-reinforced AAM (0.57) is close to double that of FA-based mortars. The efficiency of MK_2BK3b -based matrix is not only higher when compared to the FA-based TRAAM, but also to cement-based TRM. Based on preliminary tensile test results on counterpart coupons comprising a cement-based mortar of equal flexural strength to the MK_2BK3b one, the efficiency factor measured was found equal to 0.38 ^44^. The MK_2BK3b -based matrix also led to an average composite strain at peak load equal to approximately 70% of the respective strain derived from the bare textile.

### Direct shear bond tests

**Figure 5.**
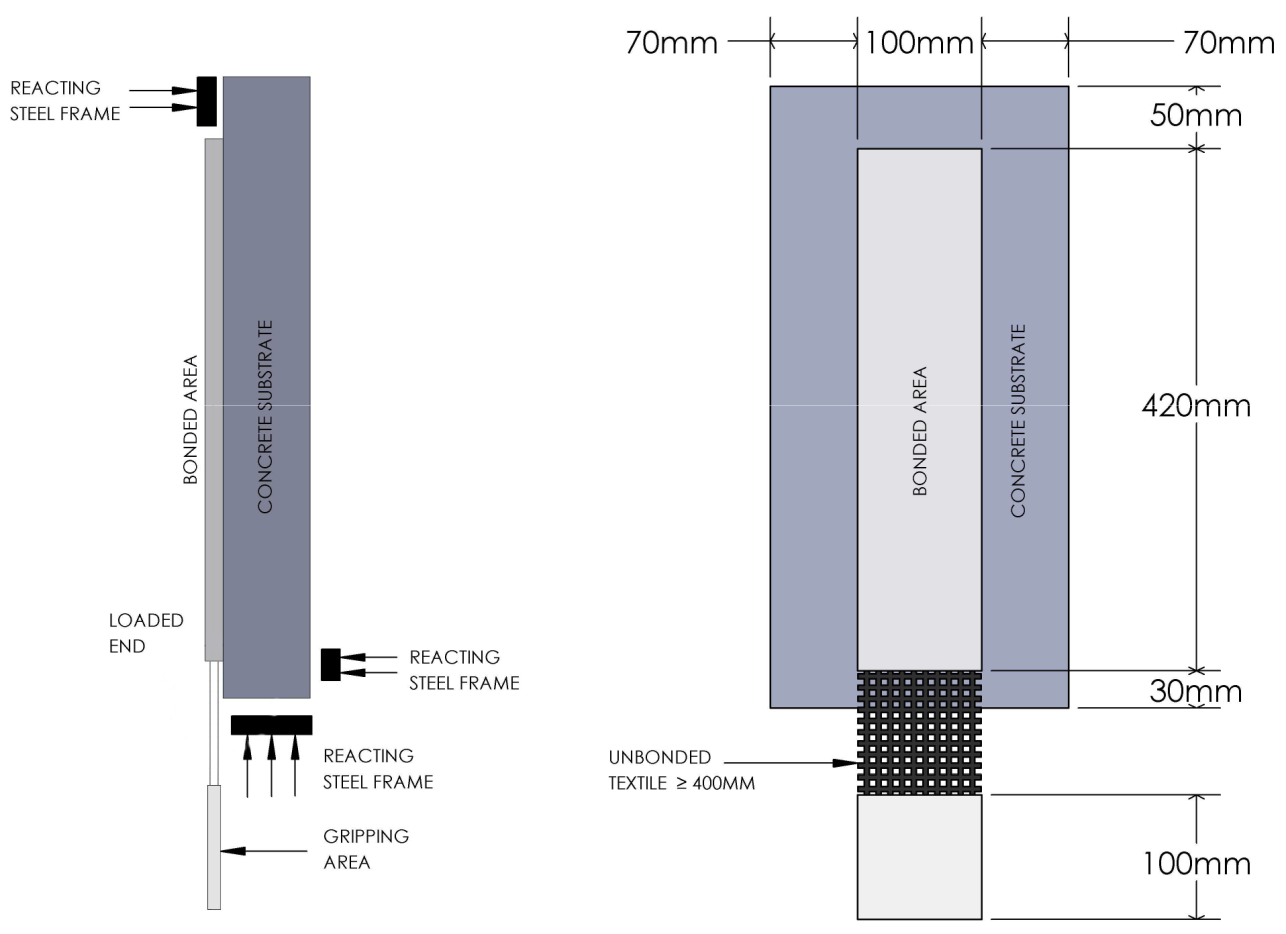
Shear bond test: specimens’ dimensions and test set up.

The TRAAM direct shear bond test followed the recommendations of the RILEM technical committee 250-CSM ^45^. The specimens’ dimensions are shown in Figure 5. The TRAAM strip had a thickness of 11–12 mm and included two layers of carbon fiber textile. The same type of carbon fiber textile as in the tensile test was used. The test aimed to quantify the bond capacity of the TRAAM strip to a concrete substrate. For the substrate, C12/16 concrete blocks of dimensions 500 mm x 240 mm x 70 mm were used. Concrete blocks were sprayed with water prior to mortar application. Spraying was repeated until the surface started to drip water instead of absorbing it. Specimens were cured for 28 days by wrapping them in wet fabrics and sealing them in plastic bags. They were tested on the same day the curing procedure ended. Figure 5 shows the single-lap shear bond test set up as executed in the laboratory. The end parts of the protruding bare carbon textile layers were epoxy-bonded to three aluminum tabs measuring 100 mm × 100 mm. The tabs helped preventing damage to the fibers by redistributing stresses along the textile. The textile was pulled at a displacement rate of 0.002 mm/s using a servo-hydraulic testing machine (MTS Systems) The metallic tabs were held by the servo-hydraulic machine upper clamp. The concrete specimen was held in place by a metallic cage which was then grabbed by the lower clamp as shown in Figure 5.

**Figure 6.**
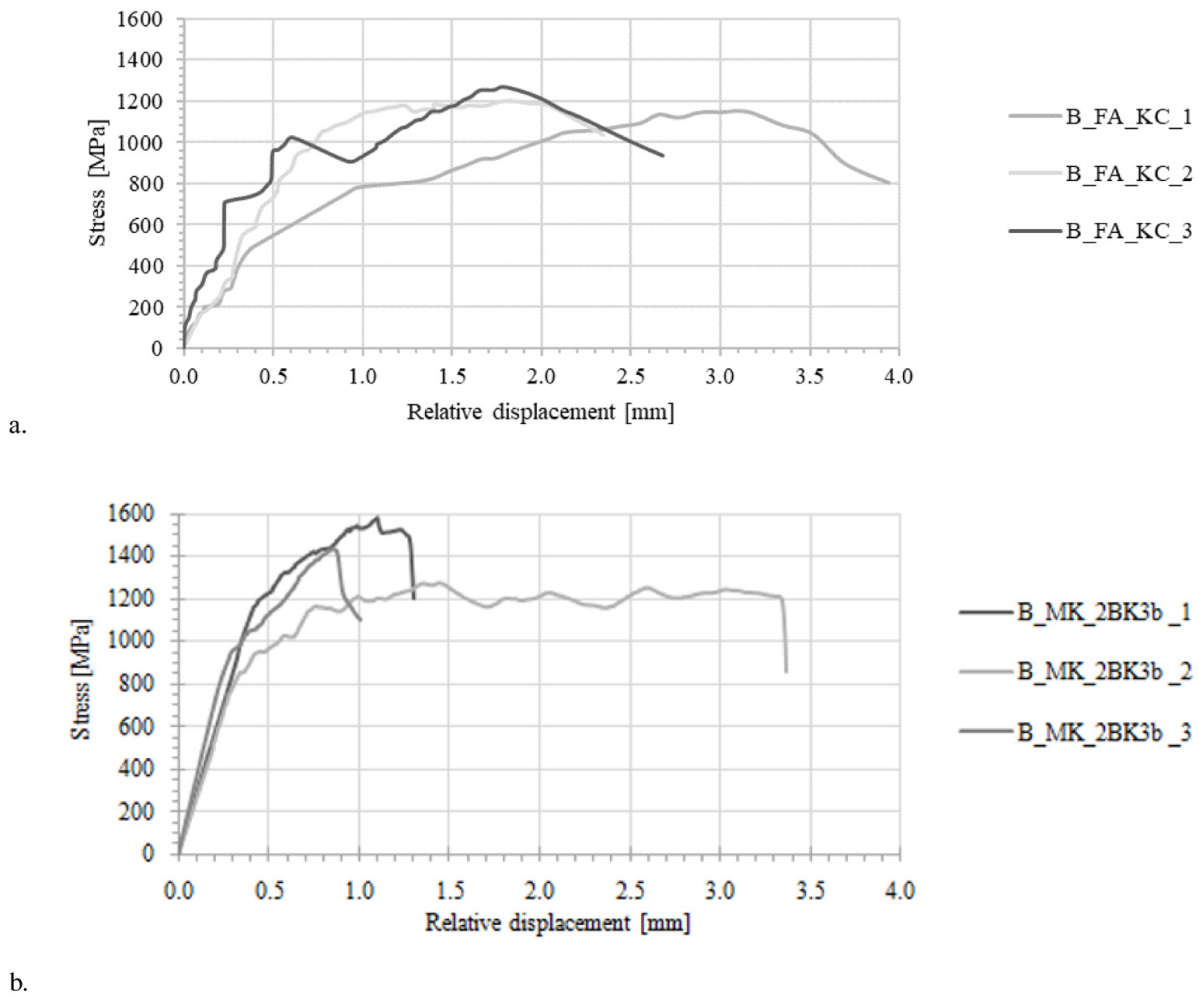
Stress-strain curves derived from shear bond tests on concrete/alkali-activated materials (TRAAM) specimens, TRAAM comprising **a**) FA_KC and **b**) MK_2BK3b mortar mixes.

The stress-strain behavior of TRAAM strips undergoing Mode II shearing off from concrete blocks is shown in Figure 6. A summary of key results is reported in Table 6. The peak stress in the composite was calculated by dividing the load by the nominal area of load-aligned fibers. Relative displacement was defined as the textile displacement in regard to the concrete substrate under the assumption that the TRAAM strip remains firmly attached on the concrete surface (zero slip of the strip in relation to the substrate). TRAAM strips comprising a FA/LFS mortar with the sodium silicate activator (B_FA_NA_i series) developed zero bond to the concrete substrate and detached even before executing the test; hence, they are not included in Table 6. TRAAM strips produced with potassium carbonate-based mortar (B_FA_KC_i series) showed a strong bond to the substrate; failure occurred due to the fibers rupturing with no signs of debonding of the mortar overlay from the concrete substrate. It is believed that failure was in this case premature owing to an uneven distribution of stress in the rovings. SEM analysis was carried out in order to shed more light on this (see next section).

**Table 6.** Results on direct shear tests of textile reinforced alkali-activated mortars bonded to a concrete surface.

Specimen ID	Peak stress (MPa)	Relative displacement at peak stress (mm)	Exploitation ratio	Average stress (MPa)	Coefficient of variation (%)	Average relative displacement at peak (mm)	Coefficient of variation (%)
B_FA_KC_1	1159.3	3.00	0.53	1216.8	4.6%	2.01	44.8%
B_FA_KC_2	1220.7	1.25	0.55				
B_FA_KC_3	1270.4	1.78	0.58				
B_MK_2BK3b_1	1581.5	1.26	0.72	1431.4	10.6%	1.83	72.7%
B_MK_2BK3b_2	1278.5	3.35	0.58				
B_MK_2BK3b_3	1434.3	0.88	0.65				

**Figure 7.**
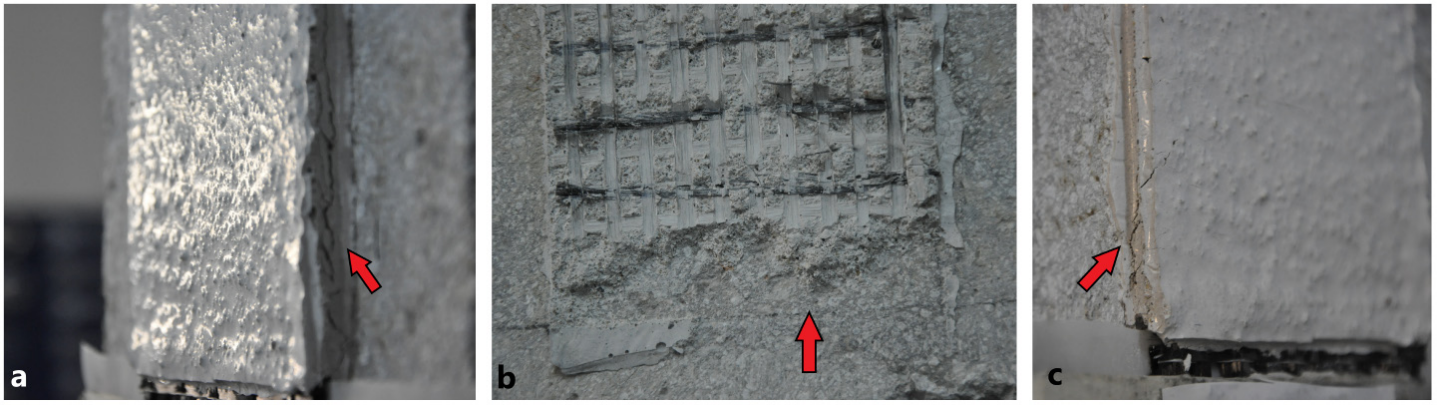
Shear bond failure types of specimens **a**) B_MK_2BK3b_1, **b**) B_MK_2BK3b_2, **c**) B_MK_2BK3b_3.

Shear bond tests on B_MK_2BK3b_i samples also showed a strong bond to the concrete substrate. The predominant cause of failure for all specimens within the group was interlaminar shear failure along the interface between the bottom mortar layer and the bottom textile strip as can be seen from Figure 7. for specimens B_MK_2BK3b_i. This was coupled with longitudinal fibers’ slippage from the mortar which was especially extensive for the case of the B_MK_2BK3b_2 specimen. Fibers’ slippage (generally, uneven between different rovings) lead to either gradual rupture of individual filaments (specimens B_MK_2BK3b_1 & B_MK_2BK3b_3) or complete detachment of the TRAAM strip along the bottom mortar layer/bottom textile strip interface (specimen B_MK_2BK3b_2) when the mortar reached its shear strength. Slippage of the longitudinal fibers – especially in specimen B_MK_2BK3b_2 – was manifested during the test by the formation of equally spaced transversal cracks along the entire length of the TRAAM strip as the transverse rovings were being pulled along the load direction through their junctions with the longitudinal ones. Although comparison between tensile and shear bond test results is rather unsound (due to differences in boundary conditions and physico-chemical interactions between the substrate and the overlay), the exploitation ratio achieved in this series of tests was slightly larger than the one achieved during tensile testing (0.65 vs. 0.57).

### Scanning electron microscopy

Evaluation of adhesion between carbon fibers and hardened matrix was performed with scanning electron microscopy (SEM) for samples from groups subjected to tensile tests. Images were obtained with a FEI QUANTA FEG 650 equipment with backscattering electrons (BSE) mode and an acceleration of 15 kV under low vacuum conditions with a working distance of 10 mm. Qualitative analysis of localized chemical composition was performed in order to visualize potential formation of specific phases in the interface of fibers with the alkali-activated matrix. For the analysis, X-ray energy dispersive spectroscopy (EDX) was used. Additional TRAAM samples were prepared specially for SEM analysis. After 28 days of curing, hydration process was stopped with solvent exchange using ethanol. Samples were then dried for two weeks and kept in a vacuum desiccator until preparation for microscopy analysis. Preparation involved vacuum impregnation with epoxy resin, water grinding with sandpaper up to 800 µm and polished up to 4 µm with a diamond paste. Ultrasonic cleaning was performed after each grinding/polishing step.

**Figure 8.**
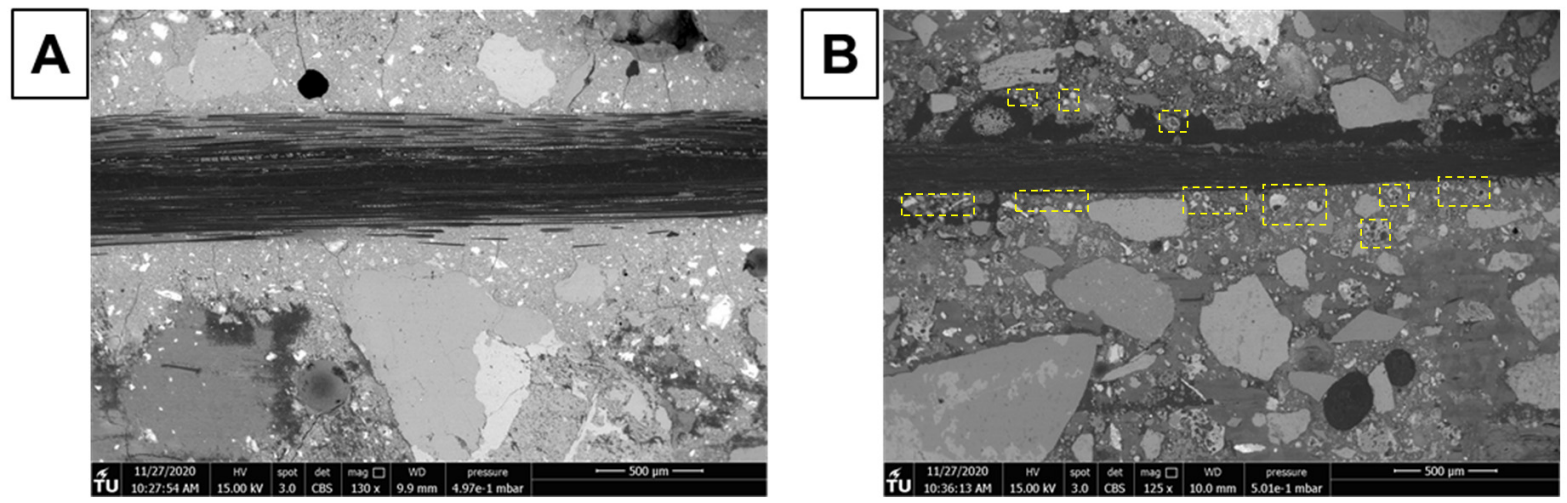
BSE images of longitudinal section of alkali-activated material samples with **a**) MK_2BK3b; and **b**) FA_NA mortar (yellow dashed squares indicate the presence of partially dissolved LFS and FA particles close to the roving).

Figure 8 shows BSE images obtained for a longitudinal section of textile reinforced mortars prepared with MK ( Figure 8.a, MK_2BK3b) and FA ( Figure 8.b, FA_NA) as main precursors, respectively. There is a clear difference in the matrix for both cases. Sample MK_2BK3b shows a homogeneous and cohesive matrix, with good adhesion of sand particles to the matrix, with no visible residual particles of undissolved MK and a small quantity of residual partially reacted LFS. Formation of such matrix is facilitated due to favored dissolution of the fine powder nature of MK ^46, 47^. Cracks along the microstructure are distributed throughout the whole image. The perpendicular orientation of these cracks to the roving indicates the occurrence of shrinkage. There is no clear evidence of differences on the distribution of particles around carbon fibers, elucidating that the adhesion between matrix and fibers was satisfactory but without formation of preferential phases in this region. The FA_NA sample displays a very heterogeneous microstructure, with presence of several partially dissolved particles of FA and LFS, as shown inside yellow dashed squares. Smaller unreacted particles of FA tend to agglomerate closer to the interface with carbon fibers. One can also notice the presence of a large continuous crack on the top matrix-fibers interface, which explains the poor performance and the absence of stage II on the stress-strain curves derived from the tensile tests ( Figure 3.a), leaving the carbon fibers as the only load-bearing component of the TRAAM.

**Figure 9.**
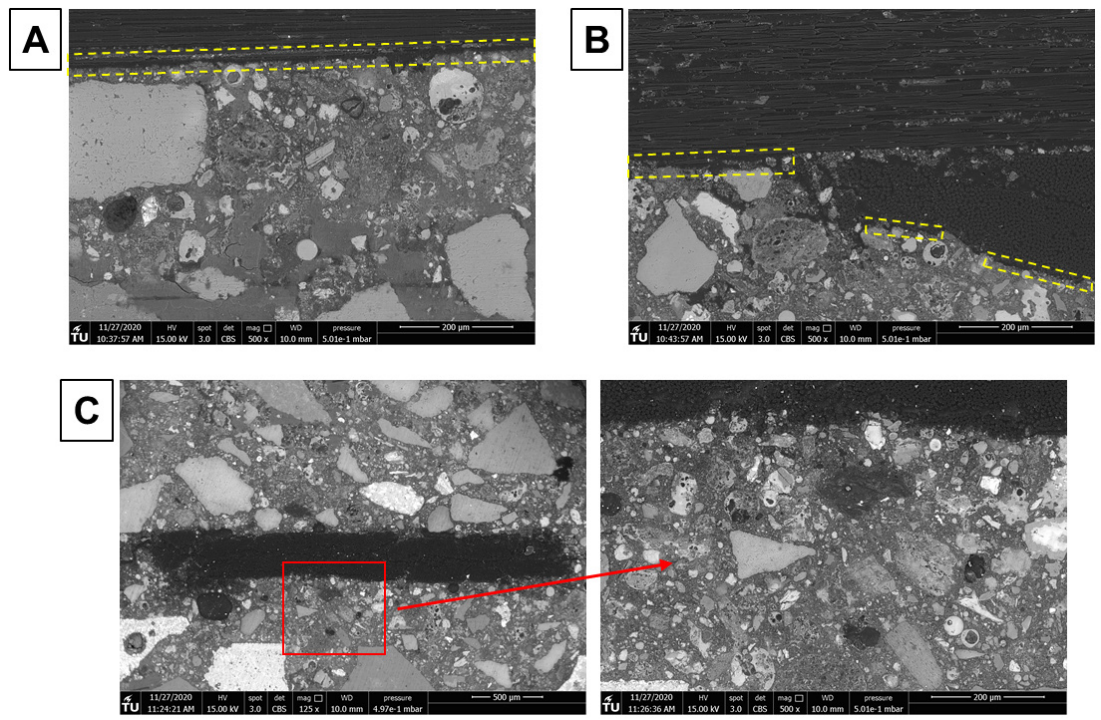
Backscattering electron images of mortars FA_NA with **a**) longitudinal and **b**) transversal carbon fibers; and **c**) FA_KC with different magnifications.

In general, mortars based on FA as main precursor presented significantly lower flexural and compressive strengths than MK-based ones, which partially explains poor mechanical test results. The molarity of activators is fairly low for such precursors, which usually demand optimum concentrations of 6–8M from NaOH/KOH solutions ^48, 49^. Although it provided proper workability during TRAAM casting, it resulted in reduced performance. It is well-known that FA displays compromised reactivity in room temperature, mainly due to its tight microstructure and low number of non-bridging oxygens (NBO) when compared to MK, and several authors reported improved reactivity and mechanical properties for MK-based binders in comparison with other low-Ca precursors for such environmental conditions ^50– 52^. As for FA-based mortars, FA_NA presented slightly higher results, with a resulting strength of 4.1 MPa, over mortar FA_KC, which displayed a resulting strength of 3.8 MPa. The fact that the latter used potassium carbonate as activator, providing lower pH to the mix, can explain such results. This is illustrated in the microstructures shown in Figure 9, in which one can identify the presence of a larger quantity of unreacted particles for mortars activated with K _2_CO _3_. The two samples showed presence of poor adhesion between matrix and fibers, as evidenced in SEM images. Figure 9.a, and Figure 9.b show, respectively, interfacial areas of hardened FA_NA binder with longitudinal and longitudinal/transversal fibers. A continuous thin crack, observed within yellow dashed squares, can be detected below the longitudinal and several unconnected regions are present surrounding the transversal fibers. As for the FA_KC binder, the amplified image shown in Figure 9.c displays a cohesive interface in between the matrix and the fibers.

**Figure 10.**
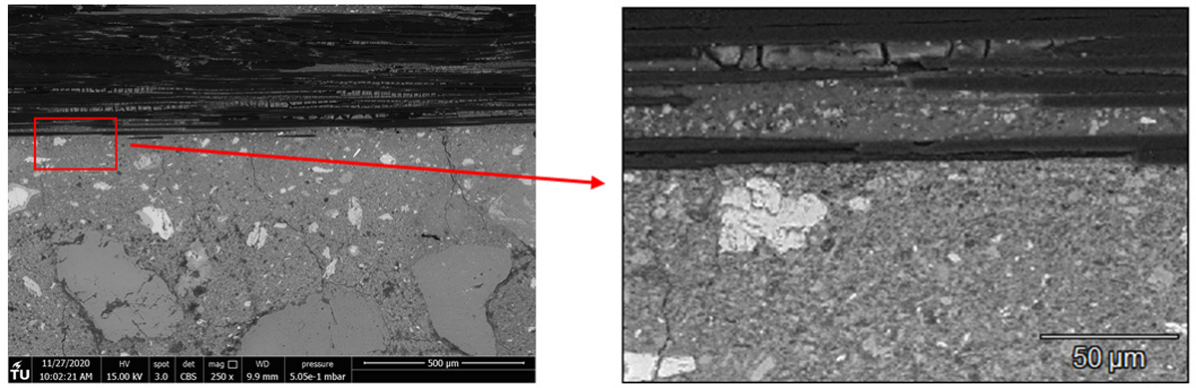
Interface closeup of carbon fibers with the MK_2BK3b hardened matrix.

**Figure 11.**
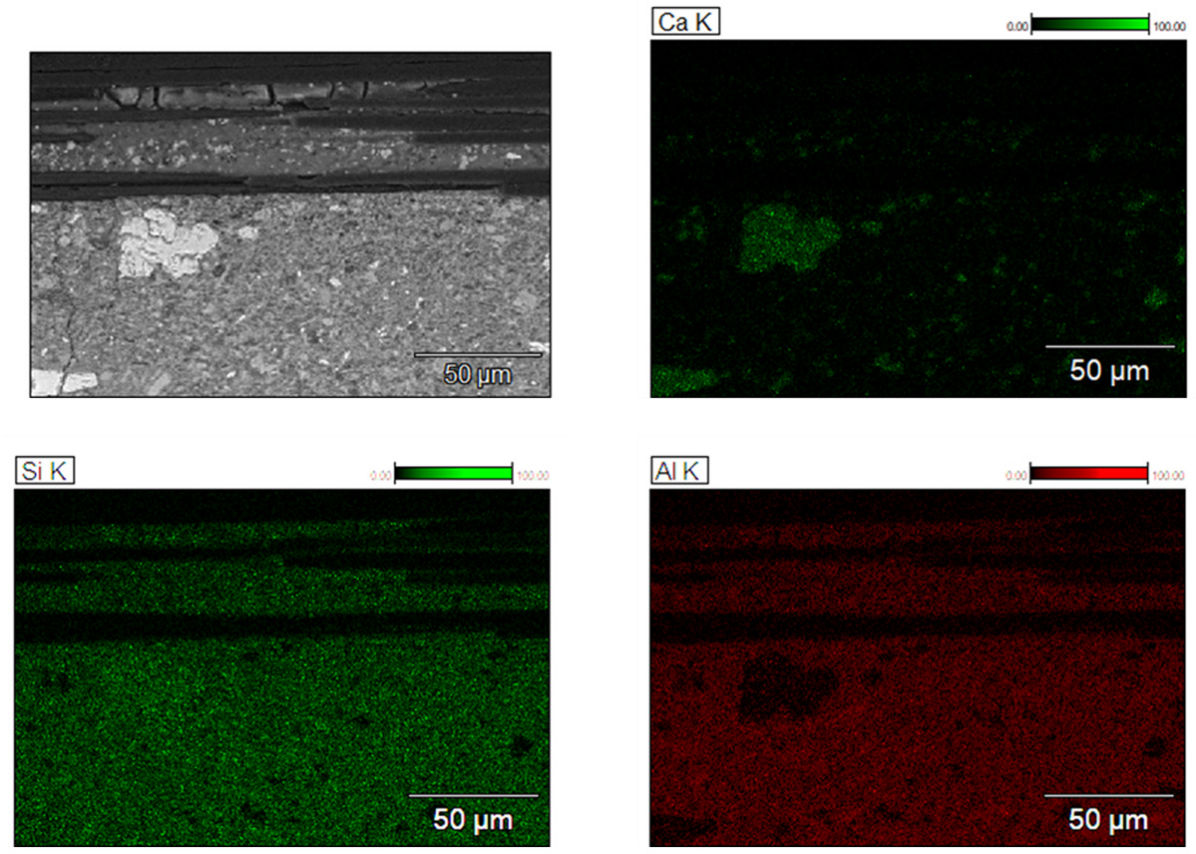
Element distribution, measured by energy dispersive X-ray spectroscopy, in different regions of hardened MK_2BK3b based mortar.

Although MK-based mortars showed the best behavior in TRAAM tensile tests, there is still room for improvement of the MK matrix-to-fibers bond efficiency. Figure 10 shows BSE images of the MK sample focused on the area below one group of longitudinal carbon fibers (roving). Underneath the roving the matrix displays several cracks and a non-continuous nature. In an attempt to visualize the potential formation of different phases in this region, Figure 11 displays the results of energy dispersive X-ray spectroscopy (EDS) analysis of the interface region of fibers with matrix. Qualitatively, no difference can be observed in the distribution of Si and Al throughout the area underneath the fibers. This is strong evidence that the formation of sodium aluminosilicate hydrate (N-A-S-H) gel is the dominant reaction mechanism in the matrix. The presence of Ca only in the regions comprising LFS confirms the absence of significant secondary reaction products in the entirety of hardened binder. As described previously in the literature, the formation of Ca-based gels, such as calcium (alumino) silicate (C-(A-)S-H) gels is not expected for low-Ca binders as MK ^50, 53^ Out of the three mortars tested for TRAAM tensile experiments, MK showed the highest fresh mortar viscosity. Hence, it can be assumed that, while the matrix had cohesive characteristics and good adhesion with the external filaments of the carbon rovings, it was not sufficiently fluid to penetrate each roving, wetting all individual fibers. Such high viscosity hinders the formation of higher interfacial area in between fibers and matrix, preventing a more improved strain hardening behavior. Other authors reported the importance of complete wetting of all individual fibers in a multifilament reinforcement in order to achieve a substantial increase in bond strength and performance ^41, 54– 56^. The higher reactivity of MK allows for good adhesion with carbon fiber rovings, at the same time it quickly decreases the fluidity of the fresh binder. These results are in agreement with experiments reported by Butler *et al.* (2010), who showed that a higher initial reactivity of the binder improves the adhesion of the matrix with fiber, enhancing the strain hardening performance of the TRM ^57^. A quantitative analysis of distribution of Si and Al might indicate that gels with different Si/Al ratios can be formed underneath the carbon fiber mesh, since this parameter affects the mobility of N-A-S-H gels and thus its ability to penetrate the tight spacing in between individual fibers. Butler *et al.* stated that the formation of rigid products adhered to reinforcement might decrease the slippage capacity of the TRM, thus leading to brittle behavior and lack of strain-hardening performance ^58^. Therefore, it is important to fully study the composition of N-A-S-H in the interface of fibers in order to design a mortar mix which can deliver enhanced properties during its whole service life.

## Conclusions

- Unprocessed high calcium FA is not recommended as the main precursor for the development of TRAAM, based on uncoated carbon textile due to relatively poor bonding capacity between matrix and fibers and/or matrix and concrete substrates.
- The FA alkali activated material and carbon textile composite depends (among other mix design and interface-related parameters) on the type of chemical activators employed. Potassium-based activators seem to outperform sodium-based ones in promoting bond. Additionally, further grinding of FA could improve its reactivity.
- A beneficial effect was observed by completely replacing silica sand with slag fine aggregate. Compressive and flexural strength increased as well as spread of the mortar. The beneficial effect in the performance indicators comes along the benefit of reusing an industry by product and thus promoting a circular economy model.
- The use of MK as the main precursor in alkali-activated mortars is very promising for the design and application of externally-bonded uncoated carbon textile reinforced composites. In this work, the best performing mix comprised a MK/LFS blend (MK_2BK3b) activated by potassium-based chemical compounds.
- The TRAAM based on MK, and uncoated carbon textile exhibited a tri-linear strain hardening behavior under uniaxial tensile loading (typical for high quality cement-based TRM) and favorable shear bond characteristics when applied on concrete surfaces. In both cases, a satisfactory textile exploitation ratio was achieved (~0.6). On the downside, MK is a processed (calcined and finely ground) natural material; hence, its use in large quantities is in opposition with the mandate of green construction practices.
- SEM imaging helped to identify causes for matrix inhomogeneity and map weak matrix-to-fiber interfaces, where present. It also confirmed that carbon fiber rovings achieved better bonding conditions when combined with the MK/LFS-based alkali-activated mortar. However, sticky consistency of the latter hindered the efficient wetting of core filaments, which calls for further strain-hardening performance improvement of MK-based mortars.
- SEM revealed some deficiencies of the MK/LFS-based mortar by identifying shrinkage cracks and unreacted particles of LFS in the mortar matrix, meaning that additional fine tuning of the mix is needed.

Finally, more work is needed in order to shed light on the long-term mechanical response and durability characteristics of TRAAM.

### Author contributions

**Andres Arce:** Conceptualization, Methodology, Formal analysis, Investigation, Writing - Original Draft, Visualization **Lazar Azdejkovic:** Methodology, Formal analysis, Investigation Writing - Original Draft, Visualization **Luiz Miranda de Lima:** Methodology, Formal analysis, Writing - Original Draft, Visualization **Catherine G. Papanicolaou:** Validation, Resources, Data Curation, Writing - Review & Editing, Visualization, Supervision, Project administration, Funding acquisition **Thanasis C. Triantafillou:** Writing - Review & Editing, Visualization, Supervision, Project administration, Funding acquisition

## Data availability

### Underlying data

Zenodo: Mechanical behavior of textile reinforced alkali-activated mortar based on fly ash, metakaolin and ladle furnace slag. https://doi.org/10.5281/zenodo.6549964 ^34^

This project contains the following underlying data:

- Fig 1 Particle size distribution of precursors and fine aggregate.csv
- Fig 2 Mortars a) Flow spread b) flexural strength and c) compressive strength.csv
- Fig 3 Stress strain curves for TRAAM specimens under uniaxial tensile loading Mortar formulations a) FA_NA b) FA_KC.csv
- Fig 6 Stress-strain curves derived from shear bond tests on concrete TRAAM specimens.csv
- Table 1 Chemical analysis of precursors, by weight.csv
- Table 2 Mix formulations based on FA and LFS precursors (quantities expressed per 1 m3 of mortar).csv
- Table 3 Mixture formulations based on previous mix SI_HI (quantities expressed per 1 m3 of mortar).csv
- Table 4 Mixes based on MK, FA and LFS blends (quantities expressed per 1 m3 of mortar).csv
- Table 5 Tensile test results of coupons made of textile reinforced alkali-activated mortars.csv
- Table 6 Results on direct shear tests of textile reinforced alkali-activated mortars bonded to a concrete surface.csv

Data are available under the terms of the Creative Commons Attribution 4.0 International license (CC-BY 4.0).

### Ethics and consent

Ethical approval and consent were not required
