# Supplementary material for: ­­­Mechanical behavior of textile reinforced alkali-activated mortar based on fly ash, metakaolin and ladle furnace slag
Source: Open Res Eur. 2022 Jun 17;2:79. [Version 1] doi: 10.12688/openreseurope.14674.1 (PMC10445836; doi:10.12688/openreseurope.14674.1)
Supplement: Peer review history

# Peer review: Reviewer response for version 1

**DOI:** 10.21956/openreseurope.15851.r30652

**Published:** 2023-01-17

**Authors:** Konstantinos Komnitsas

**Affiliations:** 1 School of Mineral Resources Engineering, Technical University of Crete, Chania, Greece

The study deals with an interesting topic, is well-designed and presented and involves several analytical techniques and in-depth analysis of the results.

 The following aspects may be further highlighted in a future version:

- Justify more clearly the degree to which the use of textile reinforced mortars (TRM) based on alkali-activated materials (AAM) is a lower carbon footprint repair solution, by also considering the raw materials used in each case.
- The quality threshold for the efficient use of recycled aggregates, which indeed offers considerable advantages.
- The effect of transport, especially over longer distances, of slag / fly ash / (meta)kaolin on the environmental impacts.
- The increased footprint from the use of metakaolin which is produced at 700-750 C.
- The potential use of different alkaline activators, with lower footprint
- Which is the anticipated effect of the use of low-Ca fly ash

Is the study design appropriate and does the work have academic merit?

Yes

Is the work clearly and accurately presented and does it cite the current literature?

Yes

If applicable, is the statistical analysis and its interpretation appropriate?

Not applicable

Are all the source data underlying the results available to ensure full reproducibility?

Yes

Are the conclusions drawn adequately supported by the results?

Yes

Are sufficient details of methods and analysis provided to allow replication by others?

Yes

Reviewer Expertise:

Waste management, life cycle analysis, hydrometallurgy, risk analysis, solid-waste decontamination

I confirm that I have read this submission and believe that I have an appropriate level of expertise to confirm that it is of an acceptable scientific standard.

# Peer review: Reviewer response for version 1

**DOI:** 10.21956/openreseurope.15851.r30118

**Published:** 2022-10-19

**Authors:** Theofanis Krevaikas

**Affiliations:** 1 Xi'an Jiaotong-Liverpool University, Suzhou, China

The paper presents the results of an experimental campaign on the tensile and single-lap shear bond behaviour of textile-reinforced mortar coupons. Three matrices were used to prepare the specimens; an alkali-activated mortar with calcium fly ash, one with metakaolin-based and one with ladle furnace slag. 

 The comments of the reviewer are summarized as follows:

- a) The literature review needs revisions since the reviewer believes it is unbalanced. It is more focused on the mortar technology, and very little information is given on the mechanical behaviour of textile-reinforced mortars with different compositions existing in the literature.
- b) The sentence ‘Despite their evident advantages……..for those materials’ is too definite for an issue such as the market operation. Please revise it.
- c) In the ‘Methods’ section and the ‘Precursors’ part ‘,…indicate a deficit in Al2O3 content for LFS and FA,….’. The authors should explain by providing the standard levels.
- d) In the ‘Mix designs’ part and ‘…an improvement in both compressive and tensile strength…’ the flexural tensile strength should replace the latter.
- e) The KC symbol in FA_KC is not explained in the same section.
- f) Table 2 needs revision since some errors in the values and the sequence of the specimens were spotted.
- g) In table 4, the ‘*’ notation is missing.
- h) In the ‘Fresh-and hardened-state properties’, the selection of the TRAAM based on ‘Although both mixes…..composite action was considered by the reviewer as abrupt and contradictory to the initial plan and set-up of the experimental objectives. An additional section in the ‘Mix Design’ or in the ‘Fresh-and hardened-state properties’ could probably be included to prepare the reader for the potential alternative.
- i) In the ‘Textile-Reinforced Alkali-activated Mortars’ sections, the authors say that ‘The experimentally obtained maximum tensile stress….’ of the textiles was 2200 MPa, but it is not clear from the text if they performed those tests or obtained those values from other sources.
- j)The use of T in the T_MK_2BK3b and of B in B_FA_KC_i is not clearly stated.
- k) What are the ‘…preferential phases in this region' when Figure 8 is explained.
- l) Figure 11 needs improvement since it is not so clear. Moreover, the images should include additional information to help the reader understand the points. However, certain information on the specific method might be included in the text to help the unfamiliar reader.               

Is the study design appropriate and does the work have academic merit?

Yes

Is the work clearly and accurately presented and does it cite the current literature?

Partly

If applicable, is the statistical analysis and its interpretation appropriate?

Not applicable

Are all the source data underlying the results available to ensure full reproducibility?

Yes

Are the conclusions drawn adequately supported by the results?

Yes

Are sufficient details of methods and analysis provided to allow replication by others?

Yes

Reviewer Expertise:

Composite materials, structural upgrading, masonry,

I confirm that I have read this submission and believe that I have an appropriate level of expertise to confirm that it is of an acceptable scientific standard.
